# Supplementary material for: Mechanisms governing the pioneering and redistribution capabilities of the non-classical pioneer PU.1
Source: Nat Commun. 2020 Jan 21;11:402. doi: 10.1038/s41467-019-13960-2 (PMC6972792; doi:10.1038/s41467-019-13960-2)
Supplement: Supplementary file 7 — Source data [file 41467_2019_13960_MOESM7_ESM.zip › Source_Data/FigureS2/FigureS2B_MotifScanOutput/KmeansCluster5/homerResults/motif27.similar.html]

motif27

## Information for motif27

A
C
G
T
G
T
A
C
A
C
G
T
A
C
G
T
G
T
A
C
G
T
C
A
A
G
T
C
C
T
G
A
C
G
A
T
A
C
T
G
C
T
A
G
A
G
C
T
T
C
A
G
T
C
A
G
  
Reverse Opposite:  

A
G
T
C
A
G
T
C
C
T
G
A
G
A
T
C
G
T
A
C
C
G
T
A
A
G
C
T
A
C
T
G
A
C
G
T
C
A
T
G
G
T
C
A
C
G
T
A
C
A
T
G
C
G
T
A
  

|  |  |
| --- | --- |
| p-value: | 1e-40 |
| log p-value: | -9.237e+01 |
| Information Content per bp: | 1.794 |
| Number of Target Sequences with motif | 68.0 |
| Percentage of Target Sequences with motif | 1.71% |
| Number of Background Sequences with motif | 53.9 |
| Percentage of Background Sequences with motif | 0.13% |
| Average Position of motif in Targets | 97.2 +/- 39.9bp |
| Average Position of motif in Background | 97.5 +/- 68.4bp |
| Strand Bias (log2 ratio + to - strand density) | 0.3 |
| Multiplicity (# of sites on avg that occur together) | 1.00 |
| Motif File: | file (matrix) reverse opposite |

### Similar de novo motifs found

|  |  |  |  |  |  |  |  |
| --- | --- | --- | --- | --- | --- | --- | --- |
| Rank | Match Score | Redundant Motif | P-value | log P-value | % of Targets | % of Background | Motif file |
| 1 | 0.917 | C G A T G A T C A C G T A C G T A G T C T C G A A G T C C T G A C G A T C A T G C T A G A G T C T C A G | 1e-34 | -79.951350 | 1.96% | 0.25% | motif file (matrix) |
| 2 | 0.865 | G A T C G T A C C G T A A G C T C T A G A G C T C T A G G T C A C G T A T C A G C G T A C G T A | 1e-31 | -71.647300 | 2.14% | 0.36% | motif file (matrix) |
| 3 | 0.759 | A T G C C A T G A G C T G A T C C T G A A G T C C T G A C G A T A C T G C T A G A G T C | 1e-28 | -65.235249 | 4.35% | 1.52% | motif file (matrix) |
